# Mid-Chain Radical Migration in the Radical Polymerization of *n*-Butyl Acrylate

**DOI:** 10.3390/polym10070765

**Published:** 2018-07-12

**Authors:** Nicholas Ballard, Antonio Veloso, José M. Asua

**Affiliations:** Polymat and Kimika Aplikatua Saila, University of the Basque Country UPV/EHU, Joxe Mari Korta Zentroa, Tolosa Etorbidea 72, Donostia/San Sebastián 20018, Spain; antonio.veloso@polymat.eu (A.V.); jm.asua@ehu.es (J.M.A.)

**Keywords:** acrylates, radical polymerization, mid-chain radical

## Abstract

The occurrence of intramolecular transfer to polymer in the radical polymerization of acrylic monomers has been extensively documented in the literature. Whilst it has been largely assumed that intramolecular transfer to polymer leads to short chain branches, there has been some speculation over whether the mid-chain radical can migrate. Herein, by the matrix-assisted laser desorption/ionization time of flight (MALDI-TOF) mass spectrometry (MS) of poly(*n*-butyl acrylate) synthesized by solution polymerization under a range of conditions, it is shown that this mid-chain radical migration does occur in the radical polymerization of acrylates conducted at high temperatures, as is evident from the shape of the molecular weight distribution. Using a mathematical model, an initial approximation of the rate at which migration occurs is made and the distribution of branching lengths formed in this scenario is explored. It is shown that the polymerizations carried out under a low monomer concentration and at high temperatures are particularly prone to radical migration reactions, which may affect the rheological properties of the polymer.

## 1. Introduction

The polymerization kinetics and final polymer microstructure of acrylic polymers produced by radical polymerization are strongly affected by transfer to polymer events that lead to the formation of a so-called mid-chain radical (MCR) [1,2,3,4]. The high stability of this radical in comparison to the end-chain radical leads to slow propagation, resulting in a decrease in the polymerization rate and a buildup of mid-chain radicals, such that the fraction of mid-chain radicals typically exceeds 0.7 [5,6,7,8,9,10]. In addition to propagation, which yields a branch point, mid-chain radicals can also undergo a *β*-scission reaction leading to a macromonomer species and a secondary radical [11,12,13,14]. This reaction is reversible; thus, mid-chain radicals can also be formed by the propagation of a secondary radical species with the macromonomer [15,16]. Due to the high activation energy of the both transfer to polymer and *β*-scission reactions, macromonomer species become increasingly present as the reaction temperature is increased [13,17,18,19,20]. The formation of branched structures and chain scission can have a huge influence on the rheological properties of acrylic polymers and therefore understanding the formation and subsequent fate of mid-chain radicals is critical in the synthesis of tailored acrylic polymers.

Transfer to polymer can occur by both intermolecular and intramolecular reactions. In the case of the more prevalent intramolecular transfer to polymer, a 1,5-hydrogen transfer reaction is the dominant pathway, which results in a mid-chain radical that is in close proximity to the end chain and thus, upon propagation, a short branch is formed. The prevalence of this transfer reaction has been demonstrated by Kajiwara, who has conducted a number of studies using the electron spin resonance (ESR) of well-defined oligomeric radicals to track the motion of the radical from the chain end to the antepenultimate monomer unit [21,22]. In contrast, intermolecular transfer to polymer, which is far less common, occurs with equal probability along the chain, and therefore propagation of the mid-chain radical formed by intermolecular transfer leads to a long chain branch. As they have a greater effect on the hydrodynamic volume of the polymer chain [23,24], long-chain branches, although less numerous than short-chain branches, play a more important role in modifying the polymer properties.

The mid-chain radical produced by intramolecular transfer can also form long-chain branches in the event that the radical migrates along the polymer backbone, which would lead to a distribution of branch lengths. If this is the case, substantial differences in the degree of chain entanglement may be expected, and therefore the rheological profile of the polymer may be altered by conducting the polymerization under conditions where mid-chain radical migration is heightened. Computational studies have shown that this migration takes place via a series of *n*:*n* + 4 hydrogen transfer reactions due to the low ring strain used to form the six-membered ring transition state [25,26]. Kajiwara has reported that such a migration can be observed in the ESR spectra of long-chain acrylates [21,27], although similar investigations using *n*-butyl acrylate (*n*-BA) have not been able to distinguish between mid-chain radical species [28]. Through the use of mass spectrometry, Vandenbergh and Junkers observed the occurrence of radical migration in products of dormant polymer chains made by controlled radical polymerization activated at high temperature in the absence of monomer [29,30]. They observed that the distribution of macromonomers produced by this reaction followed a distinct pattern which could be directly linked to the occurrence of the *n*:*n* + 4 transfer. More recently, based on the experimental work of Vandenbergh and Junkers [29,30], kinetic Monte-Carlo simulations have been used to extract estimates of the rate coefficient for radical migration [31]. However, these reactions were conducted in the absence of monomer and it remains to be seen if this reaction is significant in the presence of monomer, and if so, what the temperature range is over which radical migration can be observed. For example, it was recently reported that in the reversible addition-fragmentation chain transfer (RAFT) polymerization of *n*-BA at 60 °C, the characteristic pattern could not be observed in the distribution by MALDI-TOF [32].

Herein, results are presented that show that mid-chain radical migration occurs, albeit at relatively low rates, in the presence of monomer at temperatures above 100 °C based on the observation of unique distribution of species observed by MALDI-TOF. Reactions were conducted in solution with varying monomer concentrations and temperatures to observe how reaction conditions impact on the migration process. These experimental results are backed up by a mathematical model which predicts the distribution of individual species, including effects of radical migration along the backbone, allowing for an estimate of the rate of radical migration. The article is concluded with a brief analytical description of the propensity for radical migration under different reaction conditions, with a particular focus on the distribution of branch lengths occurring for reactions carried out at low monomer concentration (semi-batch reactions) as well as at high temperatures.

## 2. Materials and Methods

### 2.1. Materials

*n*-Butyl acrylate (*n*-BA, Quimidroga (Barcelona, Spain), technical grade) was purified by distillation and was kept at −20 °C until use. The initiators, 2,2′-azobis(2-methylpropionitrile) (AIBN, Aldrich (Steinheim, Germany), 98%) and 1,1′-Azobis(cyclohexanecarbonitrile) (ACHN, Aldrich (Steinheim, Germany), 98%), and solvent, *p*-Xylene (99%, Aldrich (Steinheim, Germany)), were used as received. All other solvents were purchased from Scharlab, were of technical grade, and were used without purification.

### 2.2. Solution Polymerization

A 1-L glass jacket reactor vessel (RTCal™, Mettler-Toledo (Greifensee, Switzerland)) equipped with an anchor impeller, platinum resistance thermometer, a nitrogen inlet and a sampling tube was used to conduct the solution polymerizations. Initially, *n*-BA (80–120 g) and *p*-Xylene (318–278 g) were added to the reactor and nitrogen was bubbled through the solution for 30 min. Then, the reaction mixture was heated to the reaction temperature under constant agitation of 200 rpm. After temperature equilibration, the initiator (either AIBN (0.12 g) for reactions conducted at 60 °C and 100 °C or ACHN (0.15 g) for reactions conducted at 140 °C) dissolved in 2 g *p*-Xylene was added in a single shot. Final conversion was measured by gravimetry and the dried polymer samples were used for subsequent analysis of molecular weight and NMR measurements.

### 2.3. SEC Measurement

The average molar mass was analyzed by size exclusion chromatography/multi angle light scattering (SEC/MALS). The equipment was composed by a LC20 pump (Shimadzu) coupled to a miniDAWN Treos multiangle (3 angles) light scattering laser photometer equipped with an He-Ne laser (λ = 658 nm), an Optilab Rex differential refractometer (λ = 658 nm) (all from Wyatt Technology Corp. (Santa Barbara, CA, USA)). Separation was carried out using one column (Styragel HR2, with pore size of 102 Å). Filtered toluene (HPLC-grade from Sigma-Aldrich (Steinheim, Germany)) was used for the calibration of the 90° angle scattering intensity. The detectors at angles other than 90° in the MALS instrument were normalized to the 90° detector using a standard (PS 28,770 g mol^−1^, Polymer Labs), which is small enough to produce isotropic scattering, at a flow rate of THF through the detectors of 1 mL min^−1^. In addition, the same standard and conditions were used to perform the alignment (interdetector delay volume) between concentration and light scattering detectors and the band broadening correction for the sample dilution between detectors.

The analysis was performed at 35 °C and THF was used as mobile phase at a flow rate of 1 mL min^−1^. The dried polymer was diluted in HPLC grade THF at concentrations of about 4 mg mL^−1^ and then was injected into the equipment. The dn dc^−1^ used for the molar mass calculation was the one corresponding to pBA (dn dc^−1^ = 0.064 mL g^−1^). The SEC/MALS data was analyzed by using the ASTRA software version 6.0.6. (Wyatt technologies, (Santa Barbara, CA, USA)). The absolute molar mass was calculated from the MALS/RI data using the Debye plot (with 1st order Zimm formalism).

### 2.4. Mass Spectrometry

MALDI-TOF MS measurements were performed on a Bruker Autoflex Speed system (Bruker, Germany) instrument equipped with a 355 nm Nd:YAG laser. All spectra were acquired in the positive-ion reflectron mode (accelerating voltage 20 kV, pressure 5 × 10^−6^ mbar). Samples were prepared with α-cyano-4-hydroxycinnamic acid (CHCA) and *trans*-2-[3-(4-*tert*-Butylphenyl)-2-methyl-2-propenylidene]malononitrile (DCTB). The CHCA matrix was dissolved at concentration of 10 g L^−1^ and DCTB at 40 g L^−1^ in THF. NaTFA was added as cationic ionization agent (approximately 10 g L^−1^ dissolved in THF). The matrix, salt and polymer solutions were premixed in the ratio 10:1:10 (matrix:salt:sample).

### 2.5. Mathematical Model

**Scheme 1:**
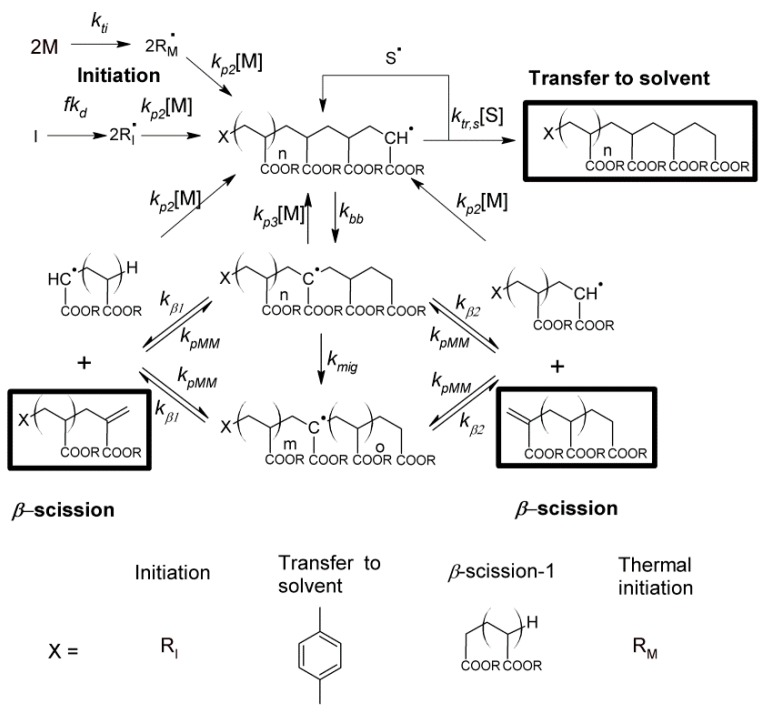
Reaction pathway in synthesis of alkyl acrylates.

In order to describe the mass distributions of the species observed in the MALDI spectra, a mathematical model was constructed according to the reaction pathway shown in Scheme 1. Equations (1)–(19) present the details of the different reactions. Equations (11)–(16) correspond to *β*-scission reactions, and the lengths of the polymer chains formed in that process depend on the position of the mid-chain radical. For the mid-chain radicals resulting from backbiting (Equations (11) and (12)), the position of the radical is given by a probability function *p*(*n*)_mig,bb_, which accounts for the migration of the MCR and is discussed in the Supplementary Materials, Appendixes I–III. For the *β*-scission of MCRs formed by intermolecular chain transfer to polymer (Equations (13) and (14)), it was assumed that the position was evenly distributed along the chain. Finally, for the MCRs formed by macromonomer propagation (Equations (15) and (16)), the position is given by the probability function *p(n)*_mig,MM_ which takes into account the initial starting position of the MCR within the chain (see Appendixes I–III in Supplementary Materials). The relative concentrations of mid-chain radicals with length *i* and starting point *j* are given by the instantaneous concentration of radicals and macromonomer species.

Initiation with thermal initiator:

(1) $$I\overset{k_{d}}{\to }2fR_{I}\;$$

Thermal initiation of *n*-BA:

(2) $$\;2M\overset{k_{ti}}{\to }2R_{M}\;$$

Propagation:

(3) $$\;R_{x}+M\overset{k_{p2}}{\to }R_{x}P_{1}$$

(4) $$\;R_{x}P_{i}+M\overset{k_{p2}}{\to }R_{x}P_{i+1}$$

Chain transfer to monomer:

(5) $$\;R_{x}P_{i}+M\overset{k_{trm}}{\to }R_{x}D_{i}+R_{M}$$

Chain transfer to solvent:

(6) $$\;R_{x}P_{i}+S\overset{k_{trs}}{\to }R_{x}D_{i}+R_{S}$$

Backbiting:

(7) $$\;R_{x}P_{i}\overset{k_{\mathrm{bb}}}{\to }R_{x}P_{i}^{\mathrm{MCR},\mathrm{bb}}$$

Intermolecular transfer to polymer:

(8) $$\;R_{x}P_{i}+R_{y}D_{j}\overset{\left(j-1\right)k_{trp}}{\to }R_{x}D_{i}+R_{y}P_{j}^{\mathrm{MCR},l}$$

Macromonomer propagation:

(9) $$\;R_{x}P_{i}+R_{y}U_{j}\overset{k_{p\mathrm{MM}}}{\to }R_{x}R_{y}P_{j+i}^{\mathrm{MCR},\mathrm{MM}}$$

Propagation of MCR:

(10) $$\;R_{x}P_{i}^{\mathrm{MCR}}+M\overset{k_{p3}}{\to }R_{x}P_{i+1}$$

*β*-scission:

(11) $$\;R_{x}P_{i}^{\mathrm{MCR},\mathrm{bb}}\overset{p{\left(n\right)}_{\mathrm{mig},\mathrm{bb}}k_{\beta 2}}{\to }R_{x}P_{i-2n-1}+R_{0}U_{2n+1}$$

(12) $$\;R_{x}P_{i}^{\mathrm{MCR},\mathrm{bb}}\overset{p{\left(n\right)}_{\mathrm{mig},\mathrm{bb}}k_{\beta 1}}{\to }R_{x}U_{i-2n}+R_{1}P_{2n}$$

(13) $$R_{x}P_{i}^{\mathrm{MCR},l}\overset{\frac{1}{i-1}k_{\beta }}{\to }R_{x}P_{i-j}+R_{0}U_{j}\;j=1,2\ldots i-1$$

(14) $$R_{x}P_{i}^{\mathrm{MCR},l}\overset{\frac{1}{i-1}k_{\beta }}{\to }R_{x}U_{i-j}+R_{1}P_{j}\;j=1,2\ldots i-1$$

(15) $$\;R_{x}P_{i}P_{j}R_{y}^{\mathrm{MCR},\mathrm{MM}}\overset{p{\left(n\right)}_{\mathrm{mig},\mathrm{MM}}k_{\beta 2}}{\to }R_{x}P_{i+j-2n-1}+R_{y}U_{2n+1}$$

(16) $$\;R_{x}P_{i}P_{j}R_{y}^{\mathrm{MCR},\mathrm{MM}}\overset{p{\left(n\right)}_{\mathrm{mig},\mathrm{MM}}k_{\beta 1}}{\to }R_{x}U_{i+j-2n}+R_{y}P_{2n}$$

Termination:

(17) $$\;R_{x}P_{i}+R_{y}P_{j}\overset{k_{t}}{\to }R_{x}R_{y}D_{i+j}$$

(18) $$\;R_{x}P_{i}^{\mathrm{MCR}}+R_{y}P_{j}\overset{k_{t}23}{\to }R_{x}D_{i}+R_{y}D_{j}$$

(19) $$R_{x}P_{i}^{\mathrm{MCR}}+R_{y}P_{j}^{\mathrm{MCR}}\overset{k_{t3}}{\to }R_{x}D_{i}+R_{y}D_{j}$$

Equations (1)–(19): Kinetic mechanism for *n*-butyl acrylate (*n*-BA) polymerization. (M: *n*-BA monomer; I: initiator; $R_{x}$: chain-end species where *x* can be *M* for monomer derived chains, *I* for initiator derived chains, *S* for chains originating from transfer to solvent, 0 or 1 for macromonomer or radical species generated from *β*-scission of tertiary radicals; $P_{i}$: chain-end radical with length *i*; $P_{i}^{\mathrm{MCR},\mathrm{bb}},\;P_{i}^{\mathrm{MCR},l}P_{i}^{\mathrm{MCR},\mathrm{MM}}$: mid-chain radical (MCR) with length *i* arising from backbiting, intermolecular chain transfer to polymer and macromonomer propagation respectively; $D_{i}$: dead polymer with length *i*; *f*: initiator efficiency factor; $U_{j}$: macromonomer with length *j*.)

Using the reaction Equations (1)–(19) above, the following material balances were used.

(20) $$\;\frac{d\left[I\right]}{dt}=\;-k_{d}\left[I\right]$$

(21) $$\frac{d\left[M\right]}{dt}=\;-k_{p2}\left[M\right]\overset{\infty }{\underset{x,i=0}{\sum }}\left[R_{x}P_{i}^{.}\right]\;-\;k_{p3}\left[M\right]\overset{\infty }{\underset{x,i=3}{\sum }}R_{x}P_{i}^{\mathrm{MCR}}\;-\;k_{trm}\left[M\right]\overset{\infty }{\underset{x,i=1}{\sum }}\left[R_{x}P_{i}^{.}\right]-2k_{ti}{\left[M\right]}^{2}\;$$

(22) $$\;\frac{d\left[R_{I}\right]}{dt}=2fk_{d}\left[I\right]\;-\;k_{p2}\left[M\right]\left[R_{I}\right]-\;k_{t}\left[R_{I}\right]\overset{\infty }{\underset{x,i=0}{\sum }}\left[R_{x}P_{i}^{.}\right]-k_{t23}\left[R_{I}\right]\overset{\infty }{\underset{x,i=3}{\sum }}\left[R_{x}P_{i}^{\mathrm{MCR}}\right]$$

(23) $$\frac{d\left[R_{M}\right]}{dt}=2k_{ti}{\left[M\right]}^{2}\;+\;k_{trm}\left[M\right]\overset{\infty }{\underset{x,i=0}{\sum }}\left[R_{x}P_{i}^{.}\right]\;-k_{p2}\left[M\right]\left[R_{M}\right]-\;k_{t}\left[R_{M}\right]\overset{\infty }{\underset{x,i=0}{\sum }}\left[R_{x}P_{i}^{.}\right]\;-k_{t23}\left[R_{M}\right]\overset{\infty }{\underset{x,i=3}{\sum }}\left[R_{x}P_{i}^{\mathrm{MCR}}\right]$$

(24) $$\frac{d\left[R_{S}\right]}{dt}=k_{trs}\left[S\right]\overset{\infty }{\underset{x,i=1}{\sum }}\left[R_{x}P_{i}^{.}\right]-k_{p2}\left[M\right]\left[R_{S}\right]-k_{t}\left[R_{S}\right]\overset{\infty }{\underset{x,i=0}{\sum }}\left[R_{x}P_{i}^{.}\right]\;-k_{t23}\left[R_{S}\right]\overset{\infty }{\underset{x,i=3}{\sum }}\left[R_{x}P_{i}^{\mathrm{MCR}}\right]$$

(25) $$\begin{array}{cc} \frac{d\left[R_{y}P_{i}^{.}\right]}{dt} & =\;k_{p2}\left[M\right]\left[R_{y}P_{i-1}^{.}\right]-k_{p2}\left[M\right]\left[R_{y}P_{i}^{.}\right]-k_{trs}\left[S\right]\left[R_{y}P_{i}^{.}\right]-\;k_{trm}\left[M\right]\left[R_{y}P_{i}^{.}\right]\\ & -k_{\mathrm{bb}}\left[R_{y}P_{i}^{.}\right]\left[i\geq 3\right]-k_{trp}\left[R_{y}P_{i}^{.}\right]\overset{\infty }{\underset{x,n=2}{\sum }}\left(n-1\right)\left[R_{x}D_{n}\right]\\ & +k_{p3}\left[M\right]\left[R_{y}P_{i-1}^{\mathrm{MCR}}\right]-k_{p\mathrm{MM}}\left[R_{y}P_{i}^{.}\right]\overset{\infty }{\underset{x,n=1}{\sum }}\left[R_{x}U_{n}\right]\\ & +k_{\beta 2}\overset{\infty }{\underset{n=1}{\sum }}\left[R_{y}P_{i+2n+1}^{\mathrm{MCR},\mathrm{bb}}\right]p{\left(n\right)}_{\mathrm{mig},\mathrm{bb}}\\ & +k_{\beta 1}p{\left(\frac{i}{2}\right)}_{\mathrm{mig},\mathrm{bb}}\overset{\infty }{\underset{x,n=i+1}{\sum }}\left[R_{x}P_{i+2n}^{\mathrm{MCR},\mathrm{bb}}\right]\left[y=1\right]\\ & +\frac{1}{2}k_{\beta }\overset{\infty }{\underset{n=i+1}{\sum }}\frac{1}{n-1}\left[R_{y}P_{n}^{\mathrm{MCR},l}\right]+\frac{1}{2}k_{\beta }\overset{\infty }{\underset{x,n=i+1}{\sum }}\frac{1}{n-1}\left[R_{x}P_{n}^{\mathrm{MCR},l}\right]\left[y=1\right]\\ & \;+k_{\beta 2}\overset{\infty }{\underset{n=1}{\sum }}\left[R_{x}P_{i+2n}^{\mathrm{MCR},MM}\right]p{\left(n\right)}_{\mathrm{mig},\mathrm{MM}}\\ & +k_{\beta 1}p{\left(\frac{i}{2}\right)}_{\mathrm{mig},\mathrm{MM}}\overset{\infty }{\underset{x,n=i+1}{\sum }}\left[R_{x}P_{i+2n}^{\mathrm{MCR},\mathrm{MM}}\right]\left[y=1\right]\\ & -k_{t}\left[R_{y}P_{i}^{.}\right]\overset{\infty }{\underset{x,n=0}{\sum }}\left[R_{x}P_{n}^{.}\right]-k_{t23}\left[R_{y}P_{i}^{.}\right]\overset{\infty }{\underset{x,n=3}{\sum }}\left[R_{x}P_{n}^{\mathrm{MCR}}\right] \end{array}$$

(26) $$\begin{array}{cc} \frac{d\left[R_{y}D_{i}\right]}{dt} & =\;k_{trs}\left[S\right]\left[R_{y}P_{i}^{.}\right]+\;k_{trm}\left[M\right]\left[R_{y}P_{i}^{.}\right]-\left(i-1\right)k_{trp}\left[R_{y}D_{i}\right]\overset{\infty }{\underset{x,n=1}{\sum }}\left[R_{x}P_{n}^{.}\right]\\ & +k_{trp}\left[R_{y}P_{i}^{.}\right]\overset{\infty }{\underset{x,n=2}{\sum }}\left(n-1\right)\left[R_{x}D_{n}\right] \end{array}$$

(27) $$\begin{array}{cc} \frac{d\left[R_{y}P_{i}^{\mathrm{MCR},\mathrm{bb}}\right]}{dt} & =k_{\mathrm{bb}}\left[R_{y}P_{i}^{.}\right]\left[i\geq 3\right]-k_{\beta }\left[R_{y}P_{i}^{\mathrm{MCR},\mathrm{bb}}\right]-k_{p3}\left[M\right]\left[R_{y}P_{i}^{\mathrm{MCR},\mathrm{bb}}\right]\\ & -k_{t23}\left[R_{y}P_{i}^{\mathrm{MCR},\mathrm{bb}}\right]\overset{\infty }{\underset{x,n=0}{\sum }}\left[R_{x}P_{n}^{.}\right]-k_{t3}\left[R_{y}P_{i}^{\mathrm{MCR},\mathrm{bb}}\right]\overset{\infty }{\underset{x,n=3}{\sum }}\left[R_{x}P_{n}^{\mathrm{MCR}}\right] \end{array}$$

(28) $$\begin{array}{cc} \frac{d\left[R_{y}P_{i}^{\mathrm{MCR},l}\right]}{dt} & =\left(i-1\right)k_{trp}\left[R_{y}D_{i}\right]\overset{\infty }{\underset{x,n=1}{\sum }}\left[R_{x}P_{n}^{.}\right]-k_{\beta }\left[R_{y}P_{i}^{\mathrm{MCR},l}\right]-k_{p3}\left[M\right]\left[R_{y}P_{i}^{\mathrm{MCR},l}\right]\\ & -k_{t23}\left[R_{y}P_{i}^{\mathrm{MCR},l}\right]\overset{\infty }{\underset{x,n=0}{\sum }}\left[R_{x}P_{n}^{.}\right]-k_{t3}\left[R_{y}P_{i}^{\mathrm{MCR},l}\right]\overset{\infty }{\underset{x,n=3}{\sum }}\left[R_{x}P_{n}^{\mathrm{MCR}}\right] \end{array}$$

(29) $$\begin{array}{cc} \frac{d\left[R_{y}P_{i}^{\mathrm{MCR},\mathrm{MM}}\right]}{dt} & =k_{pMM}\overset{\infty }{\underset{x,n=1}{\sum }}\left[R_{y}P_{i-n}^{.}\right]\left[R_{x}U_{n}\right]-k_{\beta }\left[R_{y}P_{i}^{\mathrm{MCR},\mathrm{MM}}\right]-k_{p3}\left[M\right]\left[R_{y}P_{i}^{\mathrm{MCR},\mathrm{MM}}\right]\\ & -k_{t23}\left[R_{y}P_{i}^{\mathrm{MCR},\mathrm{MM}}\right]\overset{\infty }{\underset{x,n=0}{\sum }}\left[R_{x}P_{n}^{.}\right]-k_{t3}\left[R_{y}P_{i}^{\mathrm{MCR},\mathrm{MM}}\right]\overset{\infty }{\underset{x,n=3}{\sum }}\left[R_{x}P_{n}^{\mathrm{MCR}}\right] \end{array}$$

(30) $$\begin{array}{cc} \frac{d\left[R_{y}U_{i}\right]}{dt} & =\;k_{\beta 1}\overset{\infty }{\underset{n=3}{\sum }}\left[R_{y}P_{i+2n}^{\mathrm{MCR},\mathrm{bb}}\right]p{\left(n\right)}_{\mathrm{mig},\mathrm{bb}}\\ & +k_{\beta 2}p{\left(i/2\right)}_{\mathrm{mig},\mathrm{bb}}\overset{\infty }{\underset{x,n=2i+2}{\sum }}\left[R_{x}P_{n}^{\mathrm{MCR},\mathrm{bb}}\right]\left[y=0\right]\\ & +\frac{1}{2}k_{\beta }\overset{\infty }{\underset{n=i+1}{\sum }}\frac{1}{n-1}\left[R_{y}P_{n}^{\mathrm{MCR},l}\right]+k_{\beta 1}\overset{\infty }{\underset{n=3}{\sum }}\left[R_{y}P_{i+2n}^{\mathrm{MCR},\mathrm{MM}}\right]p{\left(n\right)}_{\mathrm{mig},\mathrm{MM}}\\ & \;+\frac{1}{2}k_{\beta }\overset{\infty }{\underset{x,n=i+1}{\sum }}\frac{1}{n-1}\left[R_{x}P_{n}^{\mathrm{MCR},l}\right]\left[y=0\right]\\ & \;+k_{\beta 2}p{\left(i/2\right)}_{\mathrm{mig},\mathrm{MM}}\overset{\infty }{\underset{x,n=2i+2}{\sum }}\left[R_{x}P_{n}^{\mathrm{MCR},\mathrm{MM}}\right]\left[y=0\right]\\ & -k_{p\mathrm{MM}}\left[R_{y}U_{i}\right]\overset{\infty }{\underset{x,n=1}{\sum }}\left[R_{x}P_{n}^{.}\right] \end{array}$$

In order to limit the number of equations to be solved, macromonomer propagation reactions between species that resulted in new end-group combinations were not tracked explicitly but were joined together. In practice, this accounted for a very small number of chains in the simulations, and the small number of species detected in the MALDI spectra validates this process. In addition, a maximum chain length of 150 monomer units was used, with all species above this being grouped together.

## 3. Results

**Figure 1:**
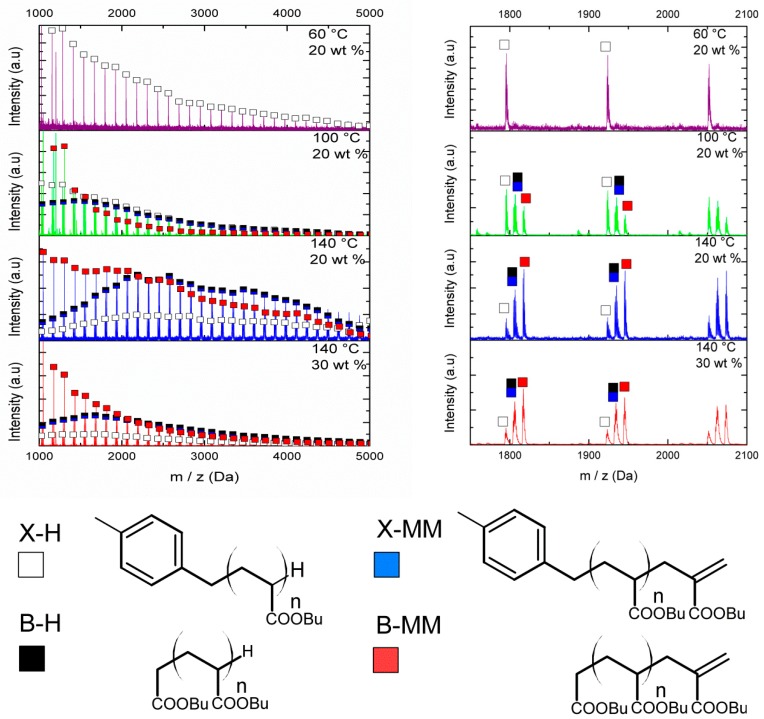
MALDI-TOF mass spectra for poly (butyl acrylate) obtained by solution polymerization at 60 °C, 100 °C or 140 °C and at 20 wt % or 30 wt % in *p*-Xylene. Enlargements in the mass range between 1700 and 2100 Da are shown in the right panel. The bottom panel shows the assigned structures of species observed in the solution polymerization of butyl acrylate in *p*-Xylene.

Solution polymerizations of butyl acrylate were conducted at different solid contents (20 wt % and 30 wt %) and at 60 °C, 100 °C and 140 °C, and the resulting polymers were analyzed by MALDI-TOF MS. A summary of the conversion, molecular weights and branching and macromonomer fractions of the final samples are given in Table 1. Due to the wide temperature range, a different initiator (ACHN) was used for the experiments conducted at 140 °C to that in the experiments conducted at 60 °C and 100 °C (AIBN). It is important to note that in all cases the polymerizations were essentially isothermal, and in no case was an exotherm greater than 5 °C observed (see Figure S1 in the Supplementary Materials). In Figure 1, the mass spectra for the samples are shown.

For reaction 1, conducted at 60 °C, a single peak was observed which was attributed to chains initiated by xylene after transfer to solvent and terminated in a hydrogen atom as a result of an additional chain transfer event (X-H in Figure 1). Due to the relatively high rate of chain transfer, the overwhelming majority of radical species are generated and terminated by transfer events, and therefore no evidence of products arising from bimolecular termination of radicals or originating from the initiator fragments was found. Comparison of the MALDI-TOF molecular weight distribution with that obtained by SEC shows that the longer polymer chains were not detected in the MALDI-TOF. This cannot be due to the relative abundance of the long chains, as shown in the number density distribution calculated from the SEC measurement of Sample 1 presented in Figure S2 in the Supplementary Materials. The difference was attributed to the lower probability of detection of high molecular weight polymers by MALDI-TOF.

Figure 1 shows that, as temperature increased, two additional groups of peaks were observed whose intensity increased at higher temperatures and where lower monomer concentration was used. The mass of these peaks corresponded to products that could be attributed to intramolecular transfer followed by *β*-scission of the mid-chain radical formed according to Scheme 1. In addition, the isotopic distribution of one of the groups of peaks revealed that it was derived from a mixture of two species of similar molecular weight (X-MM and B-H). Based on the isotopic distributions (see Figure S3 in the Supplementary Materials) and the expected products, we were able to assign the four structures as shown in Figure 1. At 100 °C, two additional groups of peaks were detected which could be assigned to chains arising from the initiator moiety and a small amount of termination by combination (see Figure S4 in the Supplementary Materials) due to the high radical flux in this particular reaction (*t*_1/2_ of AIBN at 100 °C is ≈400 s). We did not observe, in any case, low molecular weight macromonomer species, which suggests, in keeping with previous work [11,15,33], that *β*-scission tends to favor the formation of high molecular weight macromonomer and low molecular weight secondary radicals. In the low molecular weight region, MALDI-TOF analysis revealed only a small amount of butyl acrylate dimer for reactions conducted at higher temperatures, which presumably arises from thermal initiation of butyl acrylate [34,35].

It is now well documented that the majority of chains in the solution polymerization of butyl acrylate are terminated by transfer events, and the spectra in Figure 1 are to be expected based on previous studies [18,33,36]. What is surprising when we consider the spectra, however, is that not all the distributions follow the expected Flory–Schulz distribution of an exponential decay. In a free radical process, the number distribution of molecular weights would be expected to lead to an exponentially decreasing function as molecular weight increases. This is generally observed, but the peak that corresponds to B-H/X-MM species has a distinctly skewed distribution, which is not typical of a free radical process. It should be noted that the distribution given directly by MALDI-TOF does not correspond exactly to a number distribution, *N*(*M*), but rather is given by Intensity = *N*(*M*) × *M*^1/2^ [37]. In the present case, this has relatively little effect; thus, the distributions shown in Figure 1 can be approximated to a number distribution (see Figure S5 in the Supplementary Materials for a comparison of the raw data and the number distribution derived from this data).

Both the B-H and B-MM species originate from the *β*-scission of mid-chain radical species, and if we make the common assumption that intramolecular transfer occurs via a 1,5 H transfer (backbiting) and no radical migration occurs, then these species would also be expected to have the Flory distribution. While alternative intramolecular hydrogen transfer reactions are possible, experimental studies suggest the prevalence of the 1,5 backbiting reaction based on both the chain length dependency of branching density [38] and ESR experiments with short oligomers [22]. In addition, the 1,5 transfer reaction has been shown to be strongly favoured by computational studies, with alternative transfer reactions (1–7, 1–9 etc.) expected to occur several orders of magnitude slower [25].

If the mid-chain radical arises from intermolecular transfer, the products of *β*-scission would have a distribution of chain lengths which would lead to the skewed distribution observed experimentally. However, taking the recently determined values of the rate coefficient for intermolecular transfer to polymer [39], the number of mid-chain radicals generated by intermolecular transfer to polymer is far lower than those generated by intramolecular transfer under almost all conditions.

A final potential factor is the addition fragmentation process that the macromonomer species undergoes, which may give rise to a “living” chain where the number distribution forms a non-Flory–Schulz distribution and the majority of mid-chain radicals are formed by macromonomer propagation reactions. However, a shift comparable to that observed experimentally is only possible when unreasonable values of the propagation/scission rate coefficients are employed, and in that case, all the distributions are skewed (see Figure S6 in the Supplementary Materials)

**Figure 2:**
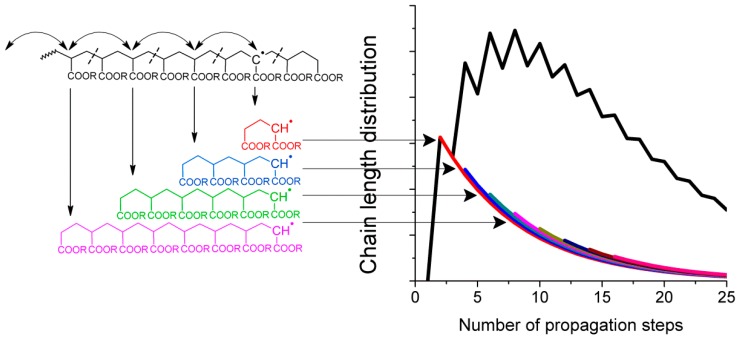
Example of hopping on molecular weight distribution. The hopping process leads to a distribution of mid-chain radicals, which after *β*-scission generates an initial distribution of chain lengths of radicals that can subsequently propagate (left). The final chain length distribution (bold black line, right) is given by the sum of the chain length distribution of each individual initiating species (colored lines, right).

Given that intramolecular transfer to polymer is the predominant cause of mid-chain radical formation, it must be this process that leads to the observed product distributions. If intramolecular transfer to polymer is followed by multiple radical migration steps along the backbone, then upon fragmentation by *β*-scission the secondary radical that initiates the new chain will have a distribution of chain lengths (see Figure 2). With each generated chain having the same final chain length distribution after propagation and termination, the sum of all the products would in turn lead to the experimentally observed distribution, as shown schematically in Figure 2.

**Scheme 2:**
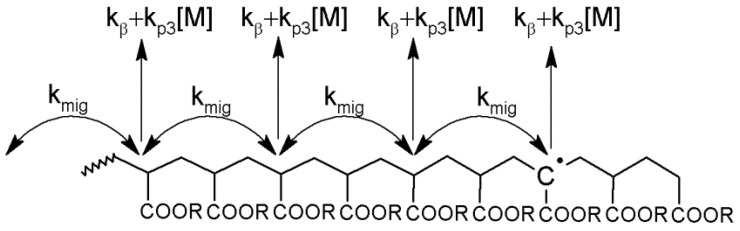
Radical migration steps starting from the initial tertiary radical position in close proximity to the end chain.

In order to further explore this idea, we constructed a mathematical model which takes into account all end group species, and incorporated the mid-chain radical migration step, assuming that migration occurs by a series of *n*:*n* + 4 H transfer reactions as suggested by theoretical studies [25]. This allows us to simulate the resulting MALDI-ToF spectrum. The effects of radical migration are based on calculation of the mid-chain radical distribution assuming the kinetic scheme laid out in Scheme 2.

The mid-chain radical formed initially in close proximity to the chain-end may either react by propagation, transfer or *β*-scission etc., or can undergo migration as seen in Scheme 2. Upon migration, there is a certain probability that the radical will return to the previous position, *P_ret_*. The mid-chain radical distribution function, *p*(*n*), which gives the probability of finding a mid-chain radical 2*n* monomer units away from the initial point of backbiting, is given by (see Appendix I in the Supplementary Materials for derivation):

(31) $$p{\left(n\right)}_{\mathrm{migration}}={\left(\frac{k_{\mathrm{mig}}\left(1-P_{ret}\right)}{k_{\mathrm{mig}}\left(1-P_{ret}\right)+k_{\beta }+k_{p3}\left[M\right]}\right)}^{n-1}\left(1-\frac{k_{mig}\left(1-P_{ret}\right)}{k_{\mathrm{mig}}\left(1-P_{ret}\right)+k_{\beta }+k_{p3}\left[M\right]}\right)\;$$

where

(32) $$\;P_{ret}=\frac{k_{\beta }+k_{p3}\left[M\right]+2k_{\mathrm{mig}}-\sqrt{{\left(k_{\beta }+k_{p3}\left[M\right]+2k_{\mathrm{mig}}\right)}^{2}-4k_{\mathrm{mig}}{}^{2}}}{2k_{\mathrm{mig}}}$$

This equation is valid on the assumption of an infinite chain length. At short chain lengths, when this assumption is no longer valid, modifications can be made to take into account the finite chain length to give a reasonable approximation of the mid-chain radical distribution function, as detailed in Appendix II of the Supplementary Materials. Finally, where the initial mid-chain radical location is not at the point closest to the end of the chain, such as may occur for MCR formation by macromonomers, the mid-chain radical distribution function may be estimated as detailed in Appendix III of the Supplementary Materials. The analytical models of mid-chain radical distribution functions are in excellent agreement with those calculated from Monte-Carlo simulations, as shown in the Supplementary Materials (Figures S7–S9).

Based on the location of the peak maximum for the simulated and the experimental MALDI spectra of experiments conducted at 140 °C, an estimation of the rate coefficient of radical migration was made, giving *k*_mig_ = 3000 s^−1^ (see Figure S9). Due to the high chain lengths in experiments at 60 and 100 degrees, it was not possible to simulate the distributions for these temperatures. All other rate coefficients were taken from literature values using those which were used to describe the kinetics, molecular weight, branching and macromonomer content of the polymerization of butyl acrylate over the same temperature range as used here (see Table 2) [20]. As no low molecular weight (*m*/*z* < 500) macromonomers were observed, the scission reaction was assumed to proceed preferentially to give low molecular weight radicals and high molecular weight macromonomer (i.e., *k_β_*_1_ > *k_β_*_2_ following Scheme 1).

**Figure 3:**
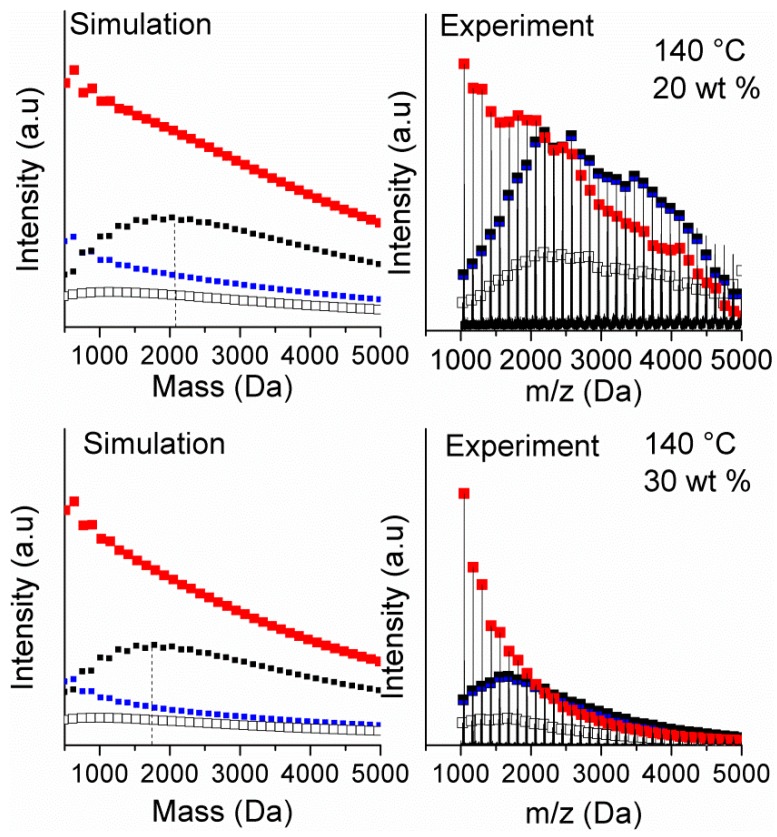
Experimental and simulated MALDI-TOF spectra at 20% (upper panel) and 30% (lower panel) and 140 °C. The experimental MALDI spectra show the complete experimental data (lines) and are overlayed with the assigned peaks using symbols corresponding to structures shown in Figure 1.

The experimental and modeled distributions at the final conversion for the reactions at 20 wt % and 30 wt % monomer concentration and 140 °C using this value are shown in Figure 3. It can be seen that, for both experiments, the experimental MALDI spectrum is reasonably described using this value of *k*_mig_. It can be seen that the inclusion of radical migration results in the B-H species displaying a skewed distribution with similar profile to the experimental spectra. Interestingly, in agreement with experimental spectra, although both B-MM and B-H originate from the scission of mid-chain radicals, the B-MM species does not present the same skewed distribution as shown by the B-H species. In both cases, initially a distribution of chain lengths of the short chain radical species is formed. For the B-H species after some number of propagation steps, the chain undergoes transfer to solvent and leads to the skewed distribution observed by MALDI. In the case of the B-MM species, the radical undergoes backbiting at some point and the tertiary radical formed can migrate. Any migration in this step acts to cancel out the migration that leads to the initial distribution of radical chain lengths. Thus, for the B-MM species a less pronounced shift away from the Flory–Schulz distribution is observed.

It should be noted that although the estimated value of *k*_mig_ is significantly higher than the recent estimate of *k*_mig_ made by Van Steenberge et al. (160 s^−1^ at 140 °C), the ratio of *k*_mig_/*k_β_* is similar in both cases (here *k*_mig_/*k_β_* = 20, whereas the value from from Van Steenberge is *k*_mig_/*k_β_* = 14). This reflects the fact that the distribution of mid-chain radicals based on the hopping mechanism is dependent on the relative rates of migration to the reaction of the mid-chain radical at each point (see Equation (31)). It is also interesting to point out that, even though it appears that *k*_mig_ >> *k_β_*, the reversible nature of migration results in the radical migrating a relatively small distance along the polymer backbone.

**Figure 4:**
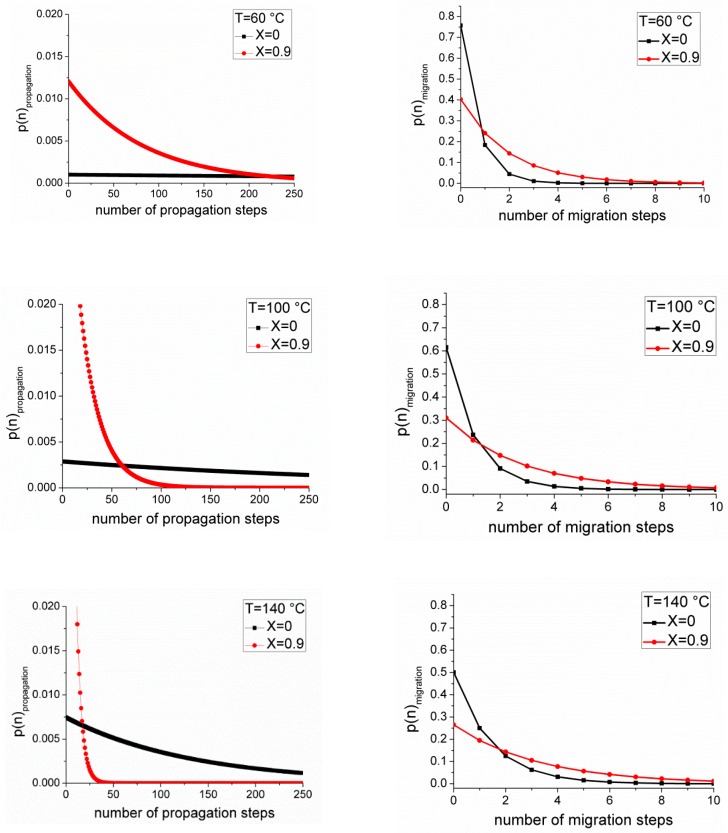
Comparison of instantaneous chain length distribution with distribution of number of radical migration steps at varying points of solution polymerization of butyl acrylate ([*M*]_0_ = 3.9 *M*) at 60 °C, 100 °C and 140 °C.

It can be seen from Figure 3 that radical migration impacts on the molecular weight distribution, but it is also worth considering the implications on the microstructure of the polymer itself. By MALDI, only those mid-chain radicals which undergo scission are seen, but a considerable number of MCRs will propagate, which leads to a distribution of branch lengths. From Equations (31) and (32), it can be seen that the number of mid-chain radical migration events is low where the rates of competing processes at the mid-chain radical center, such as mid-chain radical propagation and *β*-scission, are high. In Figure 4, the instantaneous mid-chain radical distribution function and chain length distributions for the solution polymerization of butyl acrylate calculated using Equations (A10) and (A14), respectively, from the Supplementary Materials are plotted at 60 °C, 100 °C and 140 °C at high and low monomer concentrations. The value of the rate coefficient for radical migration at 60 °C and 100 °C was estimated based on the activation energy of the backbiting reaction [25].

It can be seen from Figure 4 that at low reaction temperatures and where monomer concentration is high (low conversion), the migration of the mid-chain radical is not expected to occur significantly, and therefore only short chain branches will be present. At higher temperatures, radical migration is faster relative to propagation from the mid-chain center due to the high activation energy of H transfer reactions relative to propagation. Similarly, at low monomer concentrations, the relative rate of migration to propagation is increased, resulting in a number of migration steps before reaction. It is interesting to note that at high temperatures and low monomer concentrations, the kinetic chain length of the polymer is also lower due to the increase in transfer and *β*-scission reactions at higher temperatures. In this case, it is feasible that the mid-chain radical will be randomly distributed along the chains rather than close to the end.

Thus, while under most reaction conditions radical migration does not occur fast enough to compete with propagation (or *β*-scission) of the mid-chain center, reactions which are conducted under low monomer conditions, such as semibatch polymerizations, which are commonly performed industrially, and reactions at higher temperatures, can lead to a distribution of branch lengths. The distribution of branch lengths is important to consider because branching and branch length have subsequent consequences for the degree of chain entanglement and the viscoelastic properties of the films cast from the polymer solution and therefore radical migration may be a useful tool for tailoring physical properties of acrylic polymers.

## 4. Conclusions

In conclusion, we have shown that during the solution polymerization of butyl acrylate, mid-chain radical species are capable of migration, which leads to an unusual molecular weight distribution of certain species in the MALDI-TOF spectra. The extent of radical migration is heightened where monomer concentration is low and at higher temperatures leading to a broad distribution of branch lengths. The distribution of mid-chain radicals has been calculated analytically, which allowed for the modeling of the molecular weight distribution and gave an estimate of the rate of radical migration at high temperatures. Using these values, we have shown that, at temperatures exceeding 100 °C and at lower monomer concentrations, significant radical migration can occur that influences the microstructure and thus the rheological properties of acrylic polymers.

## Figures and Tables

**Table 1:** Summary of reactions conducted and properties of synthesized polymers.

Sample	*T* (°C)	[*M*]_0_ (mol/L)	*X*	*M_w_* (g/mol) ^a^	*Ð*	*f_br_* ^b^	*f*_MM_^c^
1	60	1.5	0.59	60,300	1.52	0.6	- ^d^
2	100	1.5	0.76	22,500	1.54	2.5	1.3
3	140	1.5	0.67	6800	1.74	4.3	2.7
4	140	2.3	0.72	13,200	1.72	3.9	1.7

^a^ Molecular weight measured by SEC-MALS; ^b^ Branching fraction measured by ^13^C NMR; ^c^ Macromonomer fraction measured by ^1^H NMR; ^d^ Concentration too low to be accurately determined by NMR.

**Table 2:** Rate coefficients used for simulation of polymerizations.

Rate Coefficient, s^−1^ or L/mol/s	Reference
$k_{d}=3.2\times 10^{17}s^{-1}\;\exp\left(-150.4\;\mathrm{kJ}.\frac{{\mathrm{mol}}^{-1}}{\mathrm{RT}}\right)$ f=0.5	[20]
$k_{p2}=2.21\times 10^{7}M^{-1}\;s^{-1}\;\exp\left(-17.9\;\mathrm{kJ}\cdot {\mathrm{mol}}^{-1}/\mathrm{RT}\right)$	[40]
$k_{trm}=2.88\times 10^{5}M^{-1}\;s^{-1}\;\exp\left(-32.6\;\mathrm{kJ}\cdot {\mathrm{mol}}^{-1}/\mathrm{RT}\right)$	[41]
$k_{trs}=k_{p}\times 1.43\times 10^{-3}$	[17]
$k_{\mathrm{bb}}=3.2\times 10^{10}{\;s}^{-1}\;\exp\left(-52.3\;\mathrm{kJ}\cdot {\mathrm{mol}}^{-1}/\mathrm{RT}\right)$	[20]
$k_{trp}=6.7\times 10^{7}M^{-1}\;s^{-1}\;\exp\left(-43.3\;\mathrm{kJ}\cdot {\mathrm{mol}}^{-1}/\mathrm{RT}\right)$	[39]
$k_{p3}=1.58\times 10^{6}M^{-1}\;s^{-1}\;\exp\left(-28.9\;\mathrm{kJ}\cdot {\mathrm{mol}}^{-1}/\mathrm{RT}\right)$	[42]
$k_{\beta }=1.47\times 10^{9}\;s^{-1}\;\exp\left(-55.4\;\mathrm{kJ}\cdot {\mathrm{mol}}^{-1}/\mathrm{RT}\right)$	[20]
$\;k_{p\mathrm{MM}}=kp_{11}\times 0.5$	[16,19]
$k_{t}=1.3\times 10^{10}M^{-1}\;s^{-1}\;\exp\left(-8.4\;\mathrm{kJ}\cdot {\mathrm{mol}}^{-1}/\mathrm{RT}\right)$	[19,43]
$k_{t23}=\sqrt{k_{t3}\times k_{t}}$	This work
$k_{t3}=1.29\times 10^{7}M^{-1}\;s^{-1}\;\exp\left(-4.0\;\mathrm{kJ}\cdot {\mathrm{mol}}^{-1}/\mathrm{RT}\right)$	[19,44]
$k_{ti}=8.27\times 10^{2}M^{-1}\;s^{-1}\;\exp\left(-73.2\;\mathrm{kJ}\cdot {\mathrm{mol}}^{-1}/\mathrm{RT}\right)$	[34]

## Supplementary Materials

The following are available online at http://www.mdpi.com/2073-4360/10/7/765/s1, Figure S1: Molecular weight distribution from SEC-MALLS and the distribution transformed to give the number distribution for Sample 1, Figure S2: Simulated and experimental MALDI-TOF mass spectra of poly(*n*-BA) synthesized at 140 °C in *p-*Xylene, Figure S3: Simulated and experimental MALDI-TOF mass spectra of the expanded region shown in the bottom figure which contains additional peaks of poly(*n*-BA) synthesized at 100 °C in *p-*Xylene (reaction 2 in Table 1), Figure S4: MALDI-TOF mass spectra (left) and number distribution calculated from raw data (right) for poly(butyl acrylate) obtained by solution polymerization at 60 °C, 100 °C or 140 °C and at 20 wt % or 30 wt % in *p-*Xylene, Figure S5: Simulated MALDI-TOF spectra with high rates of macromonomer propagation and *β*-scission at 140 °C at [*M*] = 30 wt %, Scheme S1: Radical migration steps starting from the initial tertiary radical position in close proximity to the end chain, Figure S6: Comparison of mid-chain radical distribution function based on analytical equation (lines) or kinetic Monte-Carlo simulation (symbols) for *T* = 140 °C, Figure S7: Comparison of mid-chain radical distribution function based on analytical equation (lines) or kinetic Monte-Carlo simulation (symbols) for *T* = 140 °C, Figure S8: Comparison of mid-chain radical distribution function based on analytical equation (lines) or kinetic Monte-Carlo simulation (symbols) for *T* = 140 °C, Figure S9: Simulated peak maximum for the B-H species as a function of the rate coefficient of *k*_mig_ for experiment 4 (*T* = 140 °C at [*M*] = 30 wt %, squares) and experiment 3 (*T* = 140 °C at [*M*] = 30 wt %, circles). Appendix I: Distribution of radical migration steps in the limit of long chains, Appendix II: Distribution of radical migration steps with a limiting chain length, Appendix III: Distribution of radical migration steps from a radical starting in the middle of the chain.
