# Supplementary material for: Mid-Chain Radical Migration in the Radical Polymerization of n-Butyl Acrylate
Source: Polymers (Basel). 2018 Jul 12;10(7):765. doi: 10.3390/polym10070765 (PMC6403819; doi:10.3390/polym10070765)
Supplement: Supplementary file 1 [file polymers-10-00765-s001.pdf]

SUPPORTING INFORMATION

Mid-Chain Radical Migration in the Radical Polymerization of *n*-Butyl Acrylate

Nicholas Ballard *, Antonio Veloso and José M. Asua

POLYMAT and Kimika Aplikatua Saila, University of the Basque Country UPV/EHU, Joxe Mari Korta Zentroa, Tolosa Etorbidea 72, 20018, Donostia/San Sebastián, Spain
* Correspondence: nicholas.ballard@polymat.eu; Tel.: +34-943-50-6061

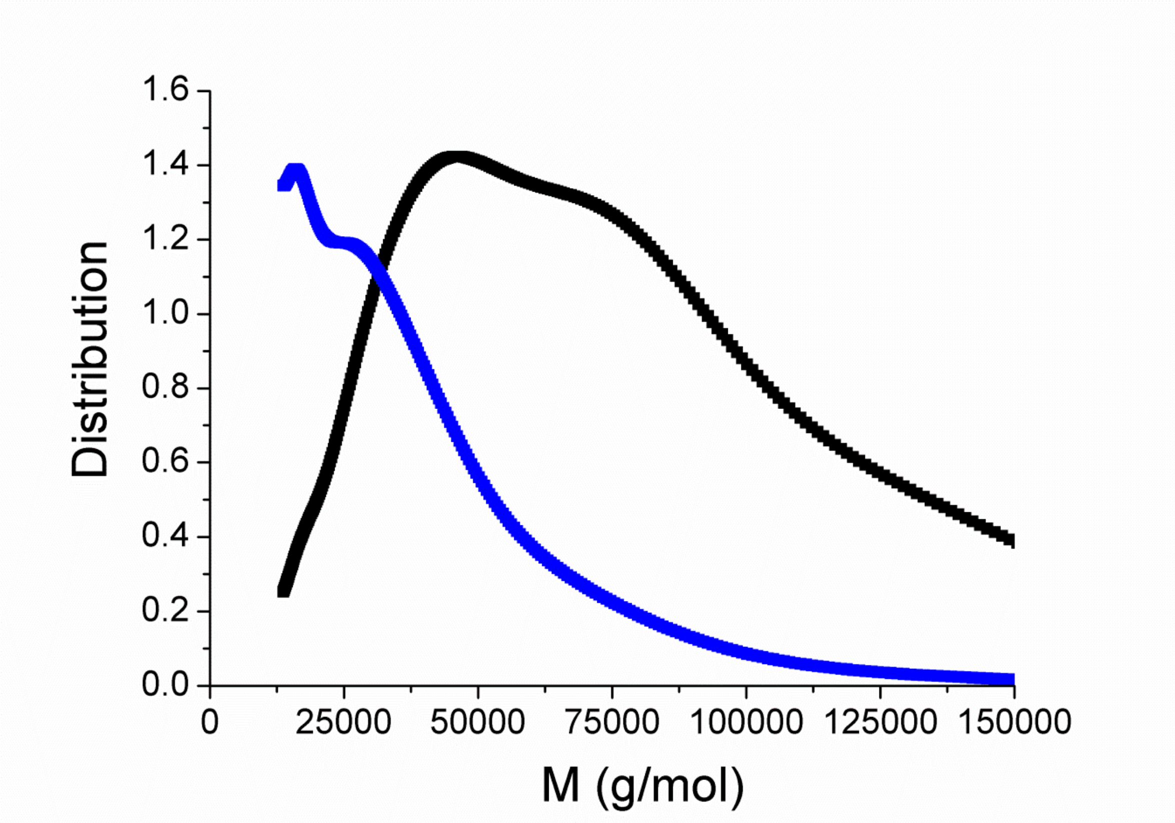

Figure S1 Molecular weight distribution from SEC-MALLS (black) and the distribution transformed to give the number distribution (blue) for Sample 1. Equipment.

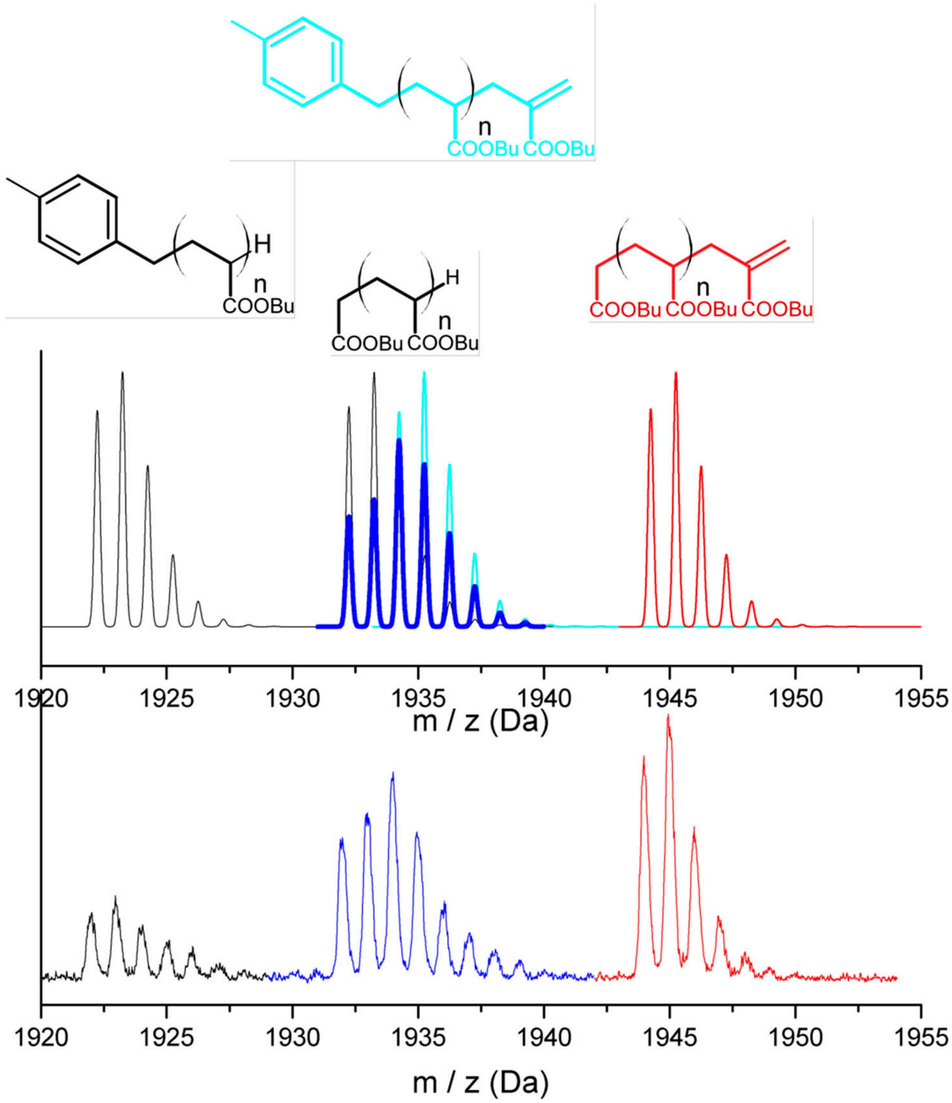

Figure S2 Simulated (top) and experimental (below) MALDI-TOF mass spectra of poly(nBA) synthesized at 140 °C in p-xylene. The second group of peaks is the simulated isotopic distribution of a 1:1 ratio of the two compounds shown above the peak (bold blue line). The individual isotopic distributions of the compounds are also shown. All peaks are the sodium adducts of the molecules shown.

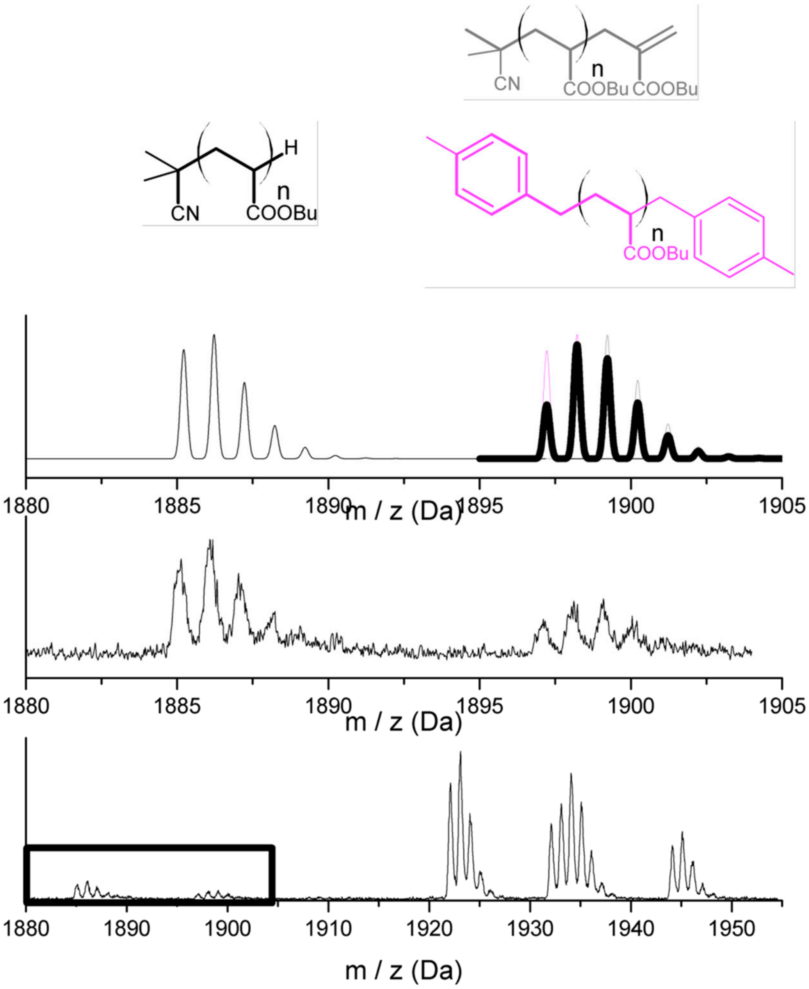

Figure S3 Simulated (top) and experimental (middle) MALDI-TOF mass spectra of the expanded region shown in the bottom figure which contains additional peaks of poly(nBA) synthesized at 100 °C in p-xylene (reaction 2 in Table S1). The second group of peaks is the simulated isotopic distribution of a 1:1 ratio of the two compounds shown above the peak (bold black line). The individual isotopic distributions of the compounds are also shown. All peaks are the sodium adducts of the molecules shown.

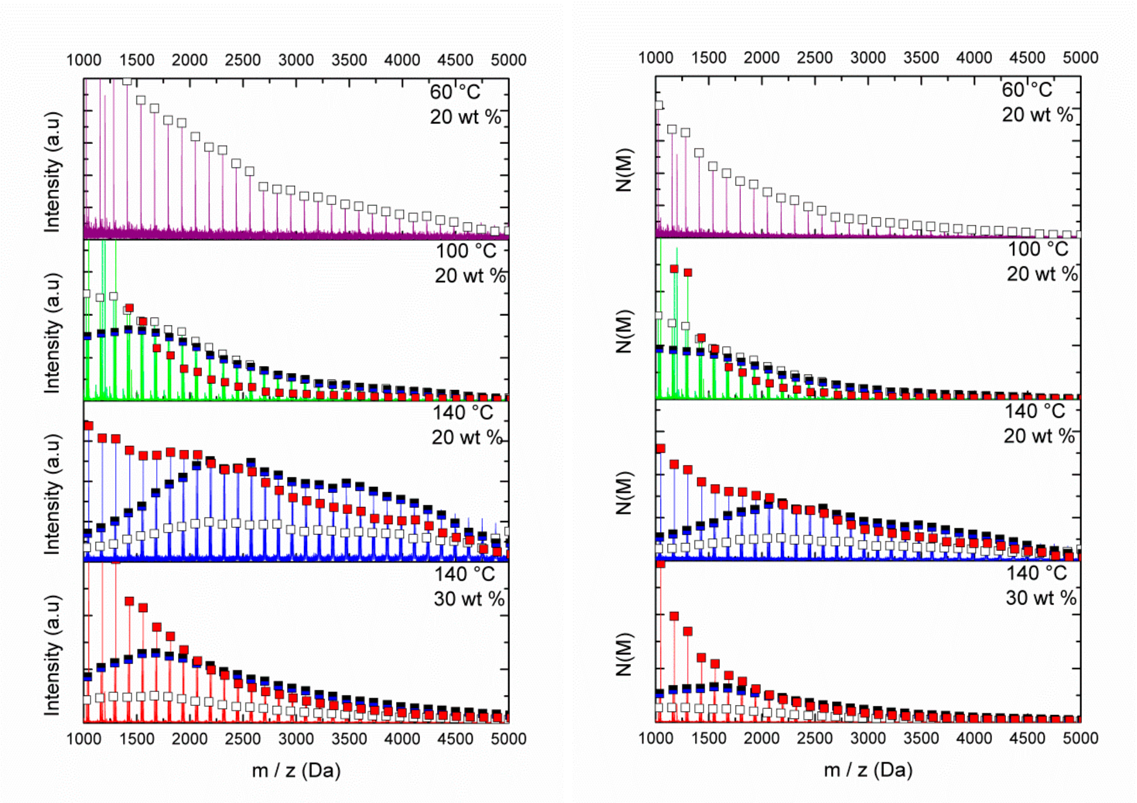

Figure S4 MALDI-TOF mass spectra (left) and number distribution calculated from raw data (right) for poly(butyl acrylate) obtained by solution polymerization at 60, 100 or 140 °C and at 20 or 30 wt% in *p*-xylene.

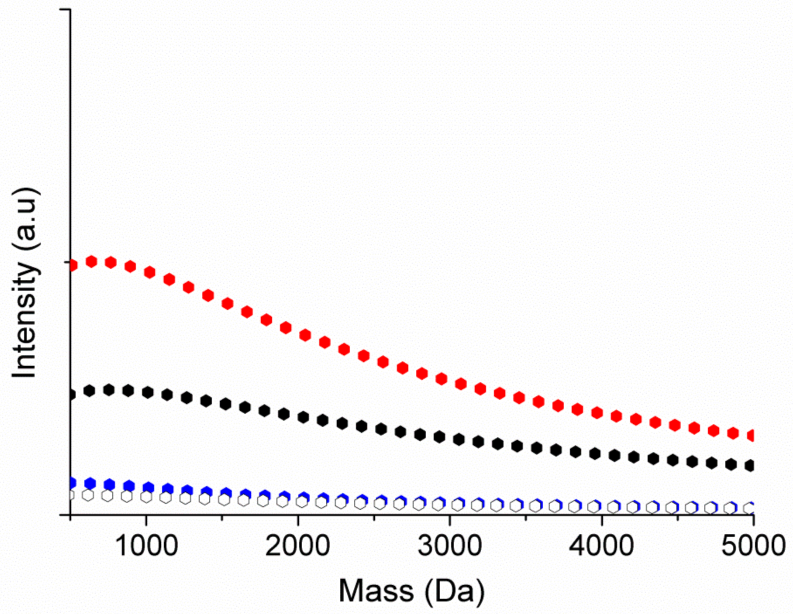

Figure S5 Simulated MALDI-TOF spectra with high rates of macromonomer propagation and β-scission at 140 °C at [M] = 30 wt%. The rate coefficients used were as shown in Table 1 with the exception of the rate coefficient of β-scission and macromonomer propagation, $k_{\beta} = 450\ s^{-1}$ $k_{pMM} = 3 \times 10^{5}\ M^{-1}s^{-1}$. Symbols correspond to structures shown in Figure 2.

Kinetic Monte Carlo simulations of midchain radical distribution

The kinetic Monte Carlo (KMC) algorithm proposed by Gillespie[1] was used as the basis of the model. Starting from an initial midchain radical located 2 units from the end of the chain three possible outcomes were considered: (1) Reaction of the midchain radical either by propagation (with frequency $1/k_{p3}[M]$) or by β-scission (with frequency $1/k_{\beta}$), (2) migration of the midchain radical two units away from the chain end (with frequency $1/k_{mig}$), (3) migration of the midchain radical two units towards the chain end (with frequency $1/k_{mig}$). In the case where the radical was next to the chain end the last reaction was disregarded. The reaction steps considered are shown in Scheme S1.

Scheme S1 Radical migration steps starting from the initial tertiary radical position in close proximity to the end chain.

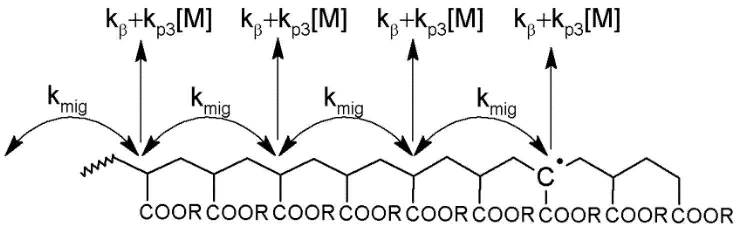

The probability of a given reaction, v, occurring, P_v, is given by:

$$P_v = \frac{R_v}{\int_{v=1}^{N} R_v}$$

Where N is the total number of reactions considered in the polymerization scheme and R_v is the rate of reaction v. At each point in the simulation a given reaction is chosen based on the above probabilities using a random number, r_1:

$\sum_{v=1}^{\mu-1} P_v < r_1 < \sum_{v=1}^{\mu} P_v$

Where μ is the number of the selected reaction. Starting from the initial location this step was repeated and the location of the midchain radical was updated until reaction of the midchain radical, either by propagation or β-scission occurred at which point the location of the midchain radical upon reaction was recorded. This was repeated for 50000 radicals in order to gain sufficient statistical information to generate the distribution function of the position at which the midchain radicals react. Figure S1 shows the comparison of the results from the Monte Carlo simulations and from the analytical model described in the main text for a variety of different conditions.

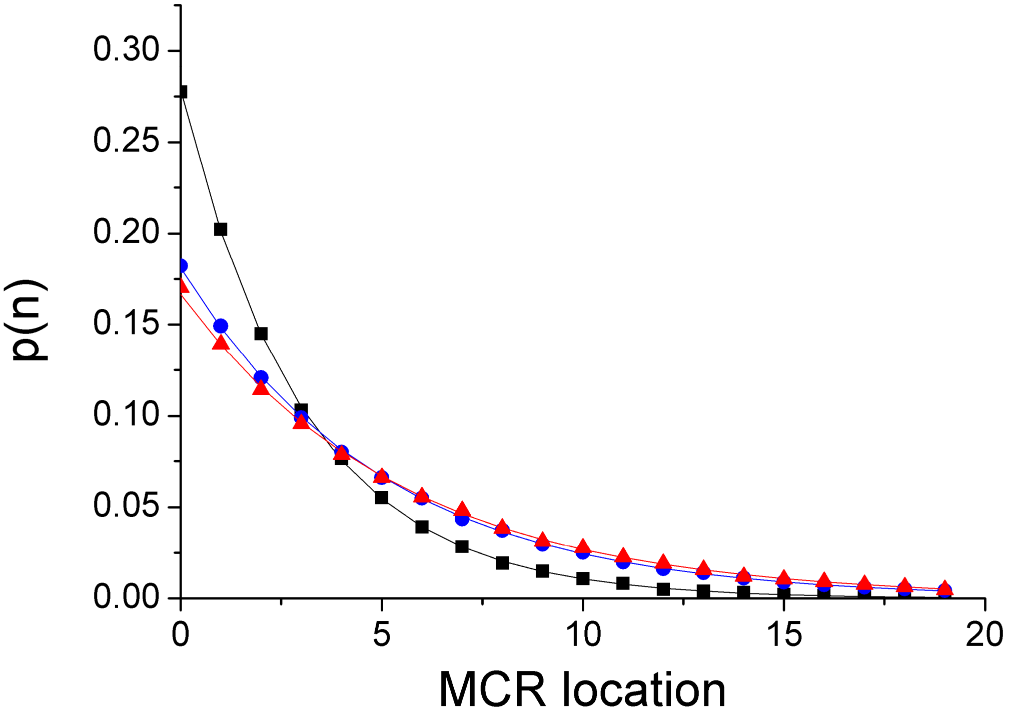

Figure S6 Comparison of midchain radical distribution function based on analytical equation (lines) or kinetic Monte-Carlo simulation (symbols) for T = 140 °C, k_{p3} = 336 M⁻

$^1s^{-1}$, k_{mig} = 4500 s^{-1}, k_β = 146 s^{-1} (a) [M] = 1 M (black squares) (b) [M] = 0.1 M (blue circles) (c) [M] = 0.01 M (red triangles).

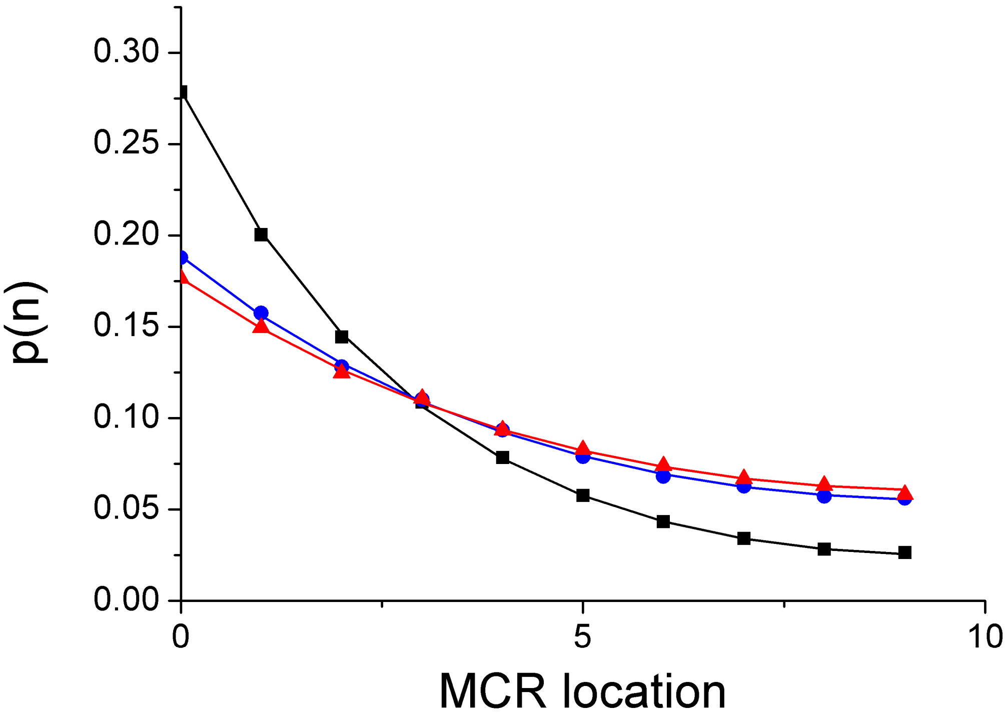

Figure S7 Comparison of midchain radical distribution function based on analytical equation (lines) or kinetic Monte-Carlo simulation (symbols) for T = 140 °C, k_{p3} = 336 $M^{-1}s^{-1}$, k_{mig} = 4500 s^{-1}, k_β = 146 s^{-1} with a maximum number of migration steps of 9 (a) [M] = 1 M (black squares) (b) [M] = 0.1 M (blue circles) (c) [M] = 0.01 M (red triangles).

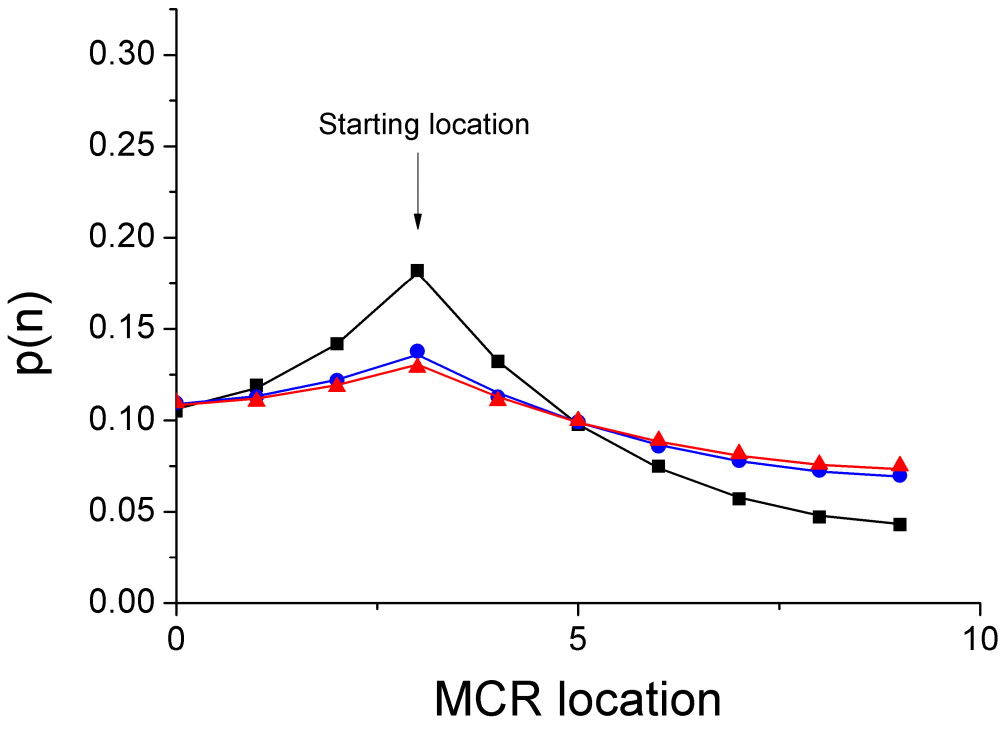

Figure S8 Comparison of midchain radical distribution function based on analytical equation (lines) or kinetic Monte-Carlo simulation (symbols) for T = 140 °C, k_{p3} = 336 $M^{-1}s^{-1}$, k_{mig} = 4500 s^{-1}, k_{β} = 146 s^{-1} with a maximum number of migration steps of 9 and starting from the middle of the polymer chain as highlighted in the figure (a) [M] = 1 M (black squares) (b) [M] = 0.1 M (blue circles) (c) [M] = 0.01 M (red triangles).

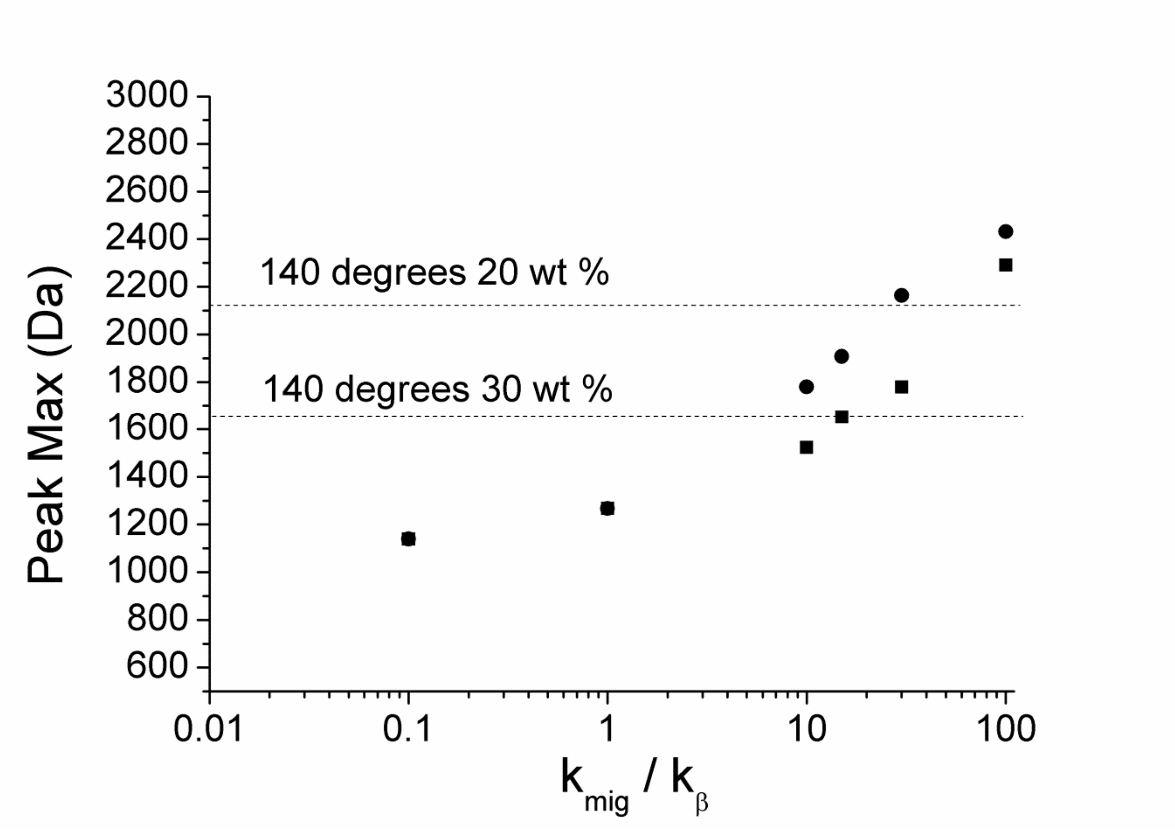

Figure S9 Simulated peak maximum for the B-H species as a function of the rate coefficient of k_{mig} for experiment 4 (T=140 °C at [M] = 30 wt%, squares) and experiment 3 (T=140 °C at [M] = 30 wt%, circles). The experimental peak maximum is shown as a dotted line.

Appendix I. Distribution of radical migration steps in the limit of long chains

In the first instance, consider a midchain radical which can either undergo an n:n+4 radical migration with a rate coefficient of k_{mig} or can undergo any alternative reaction such as propagation (with rate coefficient k_{p2}) and β-scission (with rate coefficient k_{β}), and radical migration only occurs in one direction along the chain. It is assumed that the number of migration steps is much lower than the chain length of the polymer. The probability that the radical migrates, P_m, in the first step is given by

$$P_m(1) = \frac{k_{mig}}{k_{mig}+k_{\beta}+k_{p3}[M]} \qquad \text{(A-1)}$$

The probability that the radical also migrates from the second position is given by

$$P_m(2) = P_m(1)\frac{k_{mig}}{k_{mig}+k_{\beta}+k_{p3}[M]} \qquad \text{(A-2)}$$

This leads to the general probability that the radical migrates n times

$$P_m(n) = P_m(1)^n \qquad \text{(A-3)}$$

Knowing this probability, the distribution of the number of radical migrations that occur is given by

$$p(n)_{migration} = P_m(n-1)\big(1-P_m(1)\big) \qquad \text{(A-4)}$$

This analysis does not take into account that the radical migration can occur in two directions along the chain as shown in Scheme 3. In this case, at each point the radical can either migrate in both directions or undergo reaction by β-scission or propagation. If the radical undergoes migration there is some probability that the radical will migrate back to the same position, P_{ret}. The probability that the radical migrates from the initial point is thus given by

$$P_m(1) = \frac{k_{mig}(1-P_{ret})}{k_{mig}(1-P_{ret})+k_\beta+k_{p3}[M]} \quad \text{(A-5)}$$

Where P_{ret} is the probability that the radical returns to the same point in subsequent radical migration steps given by

$$P_{ret} = \frac{k_{mig}}{B} + \frac{k_{mig}P_{ret}}{B}\frac{k_{mig}}{B} + \left(\frac{k_{mig}P_{ret}}{B}\right)^2 \frac{k_{mig}}{B} + \cdots \quad \text{(A-6)}$$

$$B = k_\beta + k_{p3}[M] + 2k_{mig} \quad \text{(A-7)}$$

Which leads to

$$P_{ret} = \frac{k_{mig}}{k_\beta+k_{p3}[M]+2k_{mig}-k_{mig}P_{ret}} \quad \text{(A-8)}$$

The value of P_{ret} can thus be calculated by

$$P_{ret} = \frac{k_\beta+k_{p3}[M]+2k_{mig}-\sqrt{\left(k_\beta+k_{p3}[M]+2k_{mig}\right)^2-4{k_{mig}}^2}}{2k_{mig}} \quad \text{(A-9)}$$

The probability that a radical migrates to the *n* position is given by Eq A-3. Thus, the distribution of the number of radical migrations that occur is given by

$$p(n)_{migration} = \left(\frac{k_{mig}(1-P_{ret})}{k_{mig}(1-P_{ret})+k_\beta+k_{p3}[M]}\right)^{n-1}\left(1-\frac{k_{mig}(1-P_{ret})}{k_{mig}(1-P_{ret})+k_\beta+k_{p3}[M]}\right) \quad \text{(A-10)}$$

Similarly, the radical chain length distribution can be given by the following. Assuming that most termination events occur either by transfer to solvent or β-scission, the probability a radical undergoes propagation is given by

$$P_{prop} = \frac{k_{p,av}[M]}{k_{p,av}[M]+k_\beta(1-P_2)+k_{tr,s}[S]P_2} \quad \text{(A-11)}$$

Where P_2 is the fraction of secondary radicals in the system which can be estimated by

$$P_2 = \frac{k_{p3}[M]}{k_{p3}[M]+k_{bb}} \quad \text{(A-12)}$$

And $k_{p,av}$ is the average propagation rate coefficient taking into account the presence of tertiary radicals, given by

$$k_{p,av} = k_{p3}(1 - P_2) + k_{p2}P_2 \quad \text{(A-13)}$$

The chain length distribution of dead chains is thus given by

$$D(n)_{propagation} = \left(\frac{k_{p,av}[M]}{k_{p,av}[M]+k_\beta(1-P_2)+k_{tr,s}[S]P_2}\right)^{n-1}\left(1-\frac{k_{p,av}[M]}{k_{p,av}[M]+k_\beta(1-P_2)+k_{tr,s}[S]P_2}\right) \quad \text{(A-14)}$$

Appendix II. Distribution of radical migration steps with a limiting chain length

When the number of migration steps is high with respect to the overall chain length, a limit to the number of migration steps must be applied in order to obtain the correct distribution. Assuming a maximum possible number of migrations steps, n_{max}, the midchain radical distribution function can be calculated as follows. The fraction of radicals that reach n_{max} migration steps is given by

$$P_m(n_{max}) = \left(\frac{k_{mig}(1-P_{ret})}{k_{mig}(1-P_{ret})+k_\beta+k_{p3}[M]}\right)^{n_{max}} \quad \text{(A-15)}$$

The midchain radical distribution function can therefore be calculated by taking this fraction of radicals and redistributing along the chain. The distribution of radicals starting from one end of the chain on the basis of the long chain hypothesis is given by

$$p(n)_{longchain,1} = \left(\frac{k_{mig}(1-P_{ret})}{k_{mig}(1-P_{ret})+k_\beta+k_{p3}[M]}\right)^{n-1}\left(1-\frac{k_{mig}(1-P_{ret})}{k_{mig}(1-P_{ret})+k_\beta+k_{p3}[M]}\right) \quad \text{(A-16)}$$

And it follows that the distribution of radicals starting from the other end of the chain at position n_{max} is given by

$$p(n)_{longchain,2} = \left(\frac{k_{mig}(1-P_{ret})}{k_{mig}(1-P_{ret})+k_\beta+k_{p3}[M]}\right)^{n_{max}-n}\left(1-\frac{k_{mig}(1-P_{ret})}{k_{mig}(1-P_{ret})+k_\beta+k_{p3}[M]}\right) \quad \text{(A-17)}$$

Thus the distribution function taking into account the limited chain length is given by

$$p(n)_{migration} = p(n)_{longchain,1} + P_m(n_{max})p(n)_{longchain,2} + P_m(n_{max})^2 p(n)_{longchain,1} + P_m(n_{max})^3 p(n)_{longchain,2} + \cdots \tag{A-18}$$

This can be simplified to give

$$p(n)_{migration} = p(n)_{longchain,1} \sum_{n=0}^{\infty}(P_m(n_{max})^2)^n + p(n)_{longchain,2} \sum_{n=0}^{\infty}\left(P_m(n_{max})\right)^n - p(n)_{longchain,2} \sum_{n=0}^{\infty}(P_m(n_{max})^2)^n \tag{A-19}$$

$$p(n)_{migration} = \frac{p(n)_{longchain,1} - p(n)_{longchain,2}}{1 - P_m(n_{max})^2} + \frac{p(n)_{longchain,2}}{1 - P_m(n_{max})} \tag{A-20}$$

Comparison of the final result with distributions obtained from Monte-Carlo simulation (see Supporting Information) confirm that this approach to estimating the midchain radical distribution is acceptable.

Appendix III. Distribution of radical migration steps from a radical starting in the middle of the chain

In some cases it may be assumed that the initial point of the midchain radical is not close to the chain end but appears at a random point in the chain. This is the case if intermolecular transfer takes place, or more importantly, in the case of midchain radical formation as a result of reaction with macromonomers.

In this case the distribution depends on the location with respect to the initial point, n_{start}. The analytical equation from above is used and divided into radicals which migrate in one direction and radicals that migrate in the other.

$$c = \left(\frac{k_{mig}(1-P_{ret})}{2k_{mig}(1-P_{ret})+k_\beta+k_{p3}[M]}\right) \qquad \text{(A-21)}$$

$$p(n)_{migration} = 1 - 2c \qquad n = n_{start} \qquad \text{(A-22)}$$

$$d = \left(\frac{k_{mig}(1-P_{ret})}{k_{mig}(1-P_{ret})+k_\beta+k_{p3}[M]}\right) \qquad \text{(A-23)}$$

$$p(n)_{migration} = cd^{(n_{start}-n-1)}(1-d) \qquad n < n_{start} \qquad \text{(A-24)}$$

$$p(n)_{migration} = cd^{(n-n_{start}-1)}(1-d) \qquad n > n_{start} \qquad \text{(A-25)}$$

The effects of limited chain length in both directions can be taken into account according to Appendix II. The probability that the radical reaches one side of the chain from the initial radical and the probability that the radical migrates the length of the chain is given by

$$P_m(n_{max,l}) = cd^{(n_{start}-1)} \qquad \text{(A-26)}$$

$$P_m(n_{max,r}) = cd^{(n_{max}-n_{start})} \qquad \text{(A-27)}$$

$$P_m(n_{max,0}) = \left(\frac{k_{mig}(1-P_{ret})}{k_{mig}(1-P_{ret})+k_\beta+k_{p3}[M]}\right)^{n_{max}} \qquad \text{(A-28)}$$

Thus the distributions can be calculated

$$p(n)_{migration} = (1-2c) + P_m(n_{max,l})\left(\frac{p(n)_{longchain,1}-p(n)_{longchain,2}}{1-P_m(n_{max})^2} + \frac{p(n)_{longchain,2}}{1-P_m(n_{max})}\right) + P_m(n_{max,r})\left(\frac{p(n_{max}-n)_{longchain,1}-p(n_{max}-n)_{longchain,2}}{1-P_m(n_{max})^2} + \frac{p(n_{max}-n)_{longchain,2}}{1-P_m(n_{max})}\right) \qquad n = n_{start} \qquad \text{(A-29)}$$

$$p(n)_{migration} = cd^{(n_{start}-n-1)}(1-d) + P_m(n_{max,l})\left(\frac{p(n)_{longchain,1}-p(n)_{longchain,2}}{1-P_m(n_{max})^2} + \frac{p(n)_{longchain,2}}{1-P_m(n_{max})}\right) + P_m(n_{max,r})\left(\frac{p(n_{max}-n)_{longchain,1}-p(n_{max}-n)_{longchain,2}}{1-P_m(n_{max})^2} + \frac{p(n_{max}-n)_{longchain,2}}{1-P_m(n_{max})}\right) \qquad n < n_{start} \qquad \text{(A-30)}$$

$$p(n)_{migration} = cd^{(n-n_{start}-1)}(1-d) + P_m(n_{max,l})\left(\frac{p(n)_{longchain,1}-p(n)_{longchain,2}}{1-P_m(n_{max})^2} + \frac{p(n)_{longchain,2}}{1-P_m(n_{max})}\right) + P_m(n_{max,r})\left(\frac{p(n_{max}-n)_{longchain,1}-p(n_{max}-n)_{longchain,2}}{1-P_m(n_{max})^2} + \frac{p(n_{max}-n)_{longchain,1}}{1-P_m(n_{max})}\right) \quad n > n_{start} \quad \text{(A-31)}$$

Comparison of the final result with distributions obtained from Monte-Carlo simulation (see Supporting Information) again confirm that this approach to estimating the midchain radical distribution is acceptable.

Reference

1. Gillespie, D. T. Exact Stochastic Simulation of Coupled Chemical Reactions *J. Phys. Chem.* **1977**, *81*, 2340–2361.
